# Experiences in rural Kenya: Addressing lack of knowledge

**Published:** 2012

**Authors:** Helen Roberts

**Affiliations:** Coordinator, Kwale District Eye Care, PO Box 90142, Mombasa, Kenya.

**Figure:**
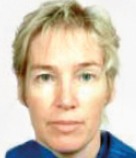
Helen Roberts

Building an eye centre in an area where you know there is great need for eye services is not enough. We began our work in Kwale district eighteen years ago after a feasibility study estimated that there were ten thousand people who were blind, thirty thousand with low vision, and about the same number with significant eye disease in the district. We knew that none of them were accessing eye care, but on our first day we saw only one eye patient!

We wanted to know why no-one was coming, so we went into the community to find out. We spoke to chiefs, attended local meetings, visited dispensaries, and went from door to door to ask if anyone knew someone who was blind. When we found a person who was blind, we asked them why they had not come.

Here are some of the answers we received:

- “People may not be able to get to your eye centre.”
- “They have no idea that you can help them.”
- “They do not understand what you are doing and therefore they are afraid.”

We realised that we had to create better awareness of who we were and what we were doing. We had to talk to people about eye health and tell them that treatment and correction of eye problems were possible. We had to show people what we were doing and what we were trying to achieve.

To do this, we trained traditional healers, women's groups, village leaders, teachers, prostitutes, whichever groups we knew people listened to. We also gave talks at community meetings and in schools and religious centres.

**Figure:**
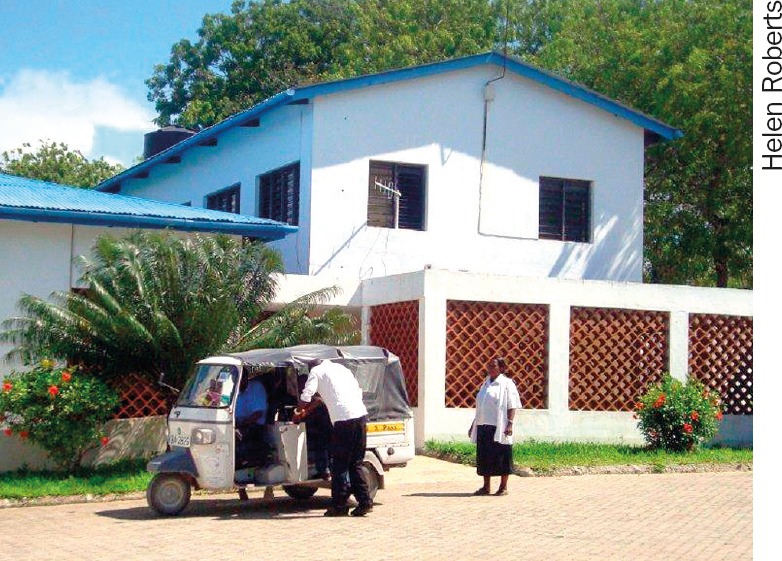
A tuk-tuk (three-wheeled taxi) now ferries patients from the main road. KENYA

At first, our scrub nurse did this work by himself between operating lists, using a bicycle to visit different areas.

Then, as the programme expanded, we recruited workers specifically for this role who were based in the community. They had to be respected members of society, literate, and able to get around. We provided a five-day training course at the eye centre, teaching them the basics about eye care and, more importantly, how to get groups of people together in the community and explain everything to them. This included information on how to get to us, how much it cost, and so on. The community workers would then find people with eye problems and arrange for our screening team to assess them in a local school or clinic. They also provided basic advice and brought those with more serious eye disease to the eye centre.

We also invited existing rural health workers, traditional birth attendants, traditional healers, and village leaders for a similar five-day workshop, one day of which would be conducted at the eye centre. Many people wanted to see for themselves that we were acting in an ethical and honest way and not, for example, removing eyes and selling them.

As well as the local population, we also needed to educate our fellow health workers in other facilities about eye care and about our specific services. In a poor rural area such as Kwale, there are many people doing many things in health. So we set up a stakeholders’ forum, now under the guidance of the ministry of health, so we could all find out about each other's activities in the field and be able to refer our patients to the right services. This may sound easy, but the work is ongoing, involving long meetings and lots of talking! Excitingly, the government of Kenya is establishing community health extension workers and community health workers in our district. We are trying to encourage these workers to come to us for primary eye care training, and we also encourage them to refer patients to us.

We ask our patients for feedback on an ongoing basis. When they requested a safe means of getting from the main road to the eye centre, we purchased a tuk-tuk (see picture) to provide a free shuttle service. Patients also asked where they could have their blood pressure checked or get help with diabetes control, so we gave a general doctor space to set up a clinic. This brings more people to the eye clinic and also helps our existing patients.

We found that the answer to lack of knowledge was to talk to all those interested and create awareness. We are still learning, but these are the most important first lessons from our project.

